# Global health diplomacy: a solution to meet the needs of disabled people in Yemen

**DOI:** 10.1186/s13031-020-00310-z

**Published:** 2020-09-24

**Authors:** Saeed Shahabi, Maryam Jalali, Kamran Bagheri Lankarani

**Affiliations:** 1 Health Policy Research Center, Institute of Health, Shiraz University of Medical Sciences, Shiraz, Iran; 2 Rehabilitation Research Center, Department of Orthotics and Prosthetics, School of Rehabilitation Sciences, Iran University of Medical Sciences, Tehran, Iran

**Keywords:** Global health diplomacy, Disabled people, Rehabilitation, Yemen

## Abstract

People with disabilities (PWD) are one of the most vulnerable groups in society during armed conflicts. According to the statistics, four million persons with disability live in Yemen. Lack of access and the use of rehabilitation services make PWD unable to retrieve their social and economic roles, which would have substantial negative impacts both on their families and community. The conflict escalation, an increase in the number the of displaced, COVID-19 pandemic, an increase in non-communicable diseases, and the exacerbation of poverty and malnutrition have rapidly enhanced the population at risk of disability in Yemen. Accordingly, effective and comprehensive approaches such as global health diplomacy (GHD) should be considered to meet the emerged needs. GHD seeks to address the common challenges in the global health system by involving all key stakeholders and establishing negotiations and diplomatic dialogue among official actors. Given the presence of various regional and international actors in Yemen and the examples of the successful use of GHD under conflict and post-conflict conditions in Iraq and Afghanistan, the use of diplomacy is crucial to respond to the needs of PWD in this war-torn country appropriately.

Since 2014, following the conflicts between the Houthis and supporters of President Abdrabbuh Mansur Hadi, Yemen experienced chaos and clashes. Furthermore, as of 25 March 2015, fighting escalated with the intervention of a Saudi-led military coalition seeking government support in Aden [1]. About 112,000 persons were killed during this conflict, of whom 12,000 were civil persons [2]. In addition, there have been many more indirect deaths as a result of disrupted health services and lack of food supplies and other key services. The over-five-year conflict has had a severe impact on public infrastructures, with 85% of the country’s population in the need of humanitarian assistance [3]. About half of the country’s health facilities are currently fully operational [4, 5]. Disastrously, using explosive weapons in crowded and populated regions is one of the unfortunate features of the war [6]. One of the biggest world’s humanitarian crisis is now occurring in Yemen.

People with disabilities (PWD) are one of the most vulnerable groups during conflicts [7]. According to the 2016 Global Burden of Disease (GBD) results, Yemen had the highest disability burden in accordance with the years lived with disability (YLDs) among 195 countries [8]. Although there is no exact statistics on the number of PWD in Yemen, it is estimated that four million persons with disability live there [4, 9]. Notably, since explosive weapons cause more severe and complex disabilities such as amputations and spinal cord injuries, the use of timely, specialized, and long-term services is crucial [10]. Meanwhile, significant damage to Yemen’s health system and infrastructures has left rehabilitation services such as physiotherapy, prosthetics and orthotics, and occupational therapy unavailable [7]. Lack of access to rehabilitation services makes PWD unable to retrieve their social and economic roles, which can have substantial negative impacts both on their families and community.

In response, several international organizations such as Humanity and Inclusion (also known as Handicap International) have initiated various programs to strengthen the rehabilitation services in Yemen [10]. Humanity and Inclusion is providing rehabilitation services to PWD and those injured through mobile and fixed disability teams, and it also operates in nine health centers to meet the needs of the vulnerable groups, especially PWD [11]. The UN Children’s Fund (UNICEF) is another active actor in this field in Yemen, with a strong focus on children with disabilities [12]. For example, over 100 children with disability across the country have received psychosocial and rehabilitation services such as physical therapy and assistive devices [12]. The International Committee of the Red Cross (ICRC) also helps PWD in Yemen by promoting physical rehabilitation centers in four cities (in Sana’a, Aden, Mukalla, and Taiz), including supplying prostheses and orthoses [13].

Despite all the efforts, the conflict escalation in recent years, the increase in the number of displaced residents [14], the COVID-19 pandemic, increases in non-communicable diseases such as Neonatal Congenital Anomalies [15], and the exacerbation of poverty and malnutrition [16] have rapidly enhanced the population at risk of disability in Yemen. Accordingly, it is necessary to respond effectively to the needs of PWD in this war-torn country by adopting effective international policies and tools such as global health diplomacy (GHD). GHD seeks to address the common challenges in the global health system by involving all key stakeholders and establishing negotiations and diplomatic dialogues among the official actors [17]. As presented in Fig. 1, GHD consists of three levels (namely core diplomacy, multi-stakeholder diplomacy, and informal diplomacy) [18].

**Fig. 1:**
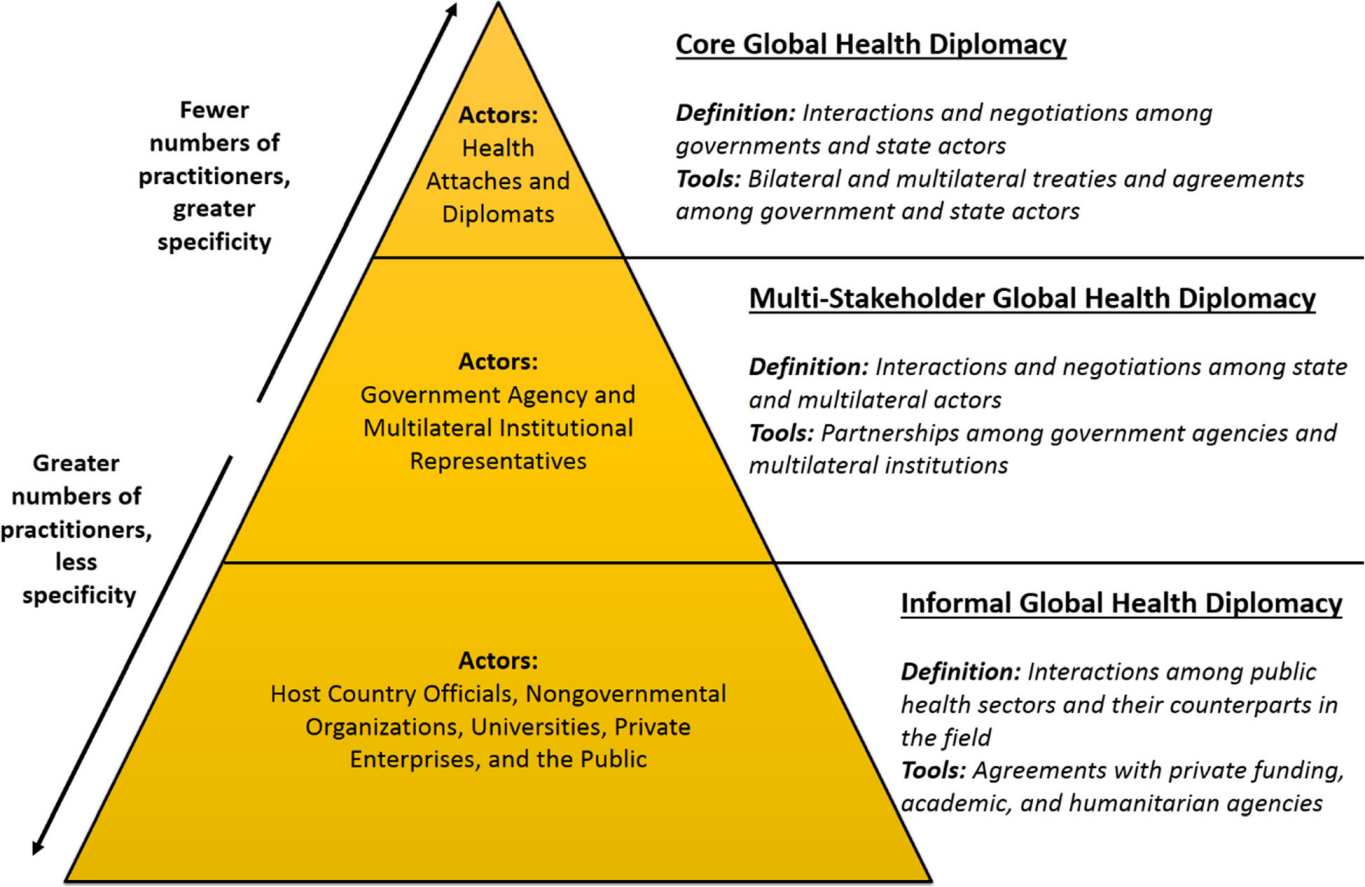
Three levels of Global Health Diplomacy (adapted from Katz et al. Defining Health Diplomacy: Changing Demands in the Era of Globalization)

Some examples of the successful use of GHD include multilateral tuberculosis programs in Iraq, in which international collaborations and diplomatic efforts led to better coverage and access to tuberculosis services [19]. A study in Afghanistan showed how GHD strengthened the implementation and delivery of the international health programs under conflict and post-conflict conditions [20]. A key element of GHD is to ensure that all targeted individuals, especially vulnerable groups such as PWD, have access to health services in crisis settings [21].

In accordance with the three levels of GHD pyramid, the core diplomacy implies negotiations and interactions among governments and international organizations to reach an agreement [18]. Therefore, addressing the challenges of PWD in Yemen at the high-level of UN and WHO meetings, such as Prevention and Control of Non-Communicable Diseases and The Paris Declaration on Aid Effectiveness [22, 23], could facilitate greater involvement of state actors and international cooperation. Furthermore, at the multi-stakeholder diplomacy level, the active participation of relevant national ministries such as the Ministry of Health, the Ministry of Foreign Affairs, academics, national research centers, and also regional organizations (such as The WHO Regional Office for the Eastern Mediterranean) is of great importance to receive advocacy, conduct research studies, hold international conferences, and influence the negotiations and policies of other countries [18]. In this regard, Rehabilitation 2030: A call for action, developed by the WHO, considers effective cooperation between stakeholders and the receipt of foreign assistance as a prerequisite for the provision of rehabilitation services to PWD, especially in poor countries [24]. In accordance with the third level of the pyramid (informal diplomacy), organizing and empowering the PWD campaigns, non-governmental organizations, and other private institutions as well as proper interaction with foreign counterparts can be a stimulus to this dimension [18]. Campaigns against alcohol and tobacco consumption are some successful examples in this regard [23].

Numerous meetings and summits on Yemen are held annually worldwide to try and establish peace and respond to the needs of individuals as incorporating the PWD-related issues in diplomatic negotiations can facilitate an optimal response to the rising needs. However, increased awareness about the challenges of PWD and potential solutions is also needed among diplomats and others involved in such efforts. Rehabilitation experts, relevant non-governmental organizations, and patient councils must be involved in meetings to provide a clear picture of the needs of PWD in conflicts such as Yemen. In general, international collaboration via active health diplomacy is required to deal with the PWD challenges in war-torn Yemen.

## Data Availability

Not applicable.

## Abbreviations

GBD: Global Burden of Disease
YLDs: Years lived with disability
UNICEF: United Nations International Children’s Emergency Fund
ICRC: International Committee of the Red Cross
GHD: Global Health Diplomacy
